# Locomotor training using an overground robotic exoskeleton in long-term manual wheelchair users with a chronic spinal cord injury living in the community: *Lessons learned from a feasibility study in terms of recruitment, attendance, learnability, performance and safety*

**DOI:** 10.1186/s12984-018-0354-2

**Published:** 2018-03-01

**Authors:** Dany H. Gagnon, Manuel J. Escalona, Martin Vermette, Lívia P. Carvalho, Antony D. Karelis, Cyril Duclos, Mylène Aubertin-Leheudre

**Affiliations:** 1 School of Rehabilitation, Université de Montréal, Montreal, QC Canada; 2 Pathokinesiology Laboratory, Centre for Interdisciplinary Research in Rehabilitation of Greater Montreal, Centre intégré universitaire de santé et services sociaux du Centre-Sud-de-l’Île-de-Montréal, Installation Institut de réadaptation Gingras-Lindsay-de-Montréal, 6300 Avenue Darlington, Montreal, QC H3S 2J4 Canada; 3 Department of Exercise Science, Université du Québec à Montréal, Montreal, QC Canada

**Keywords:** Exercise, Paraplegia, Physical medicine and rehabilitation, Robotics, Therapies, Technology, Walking

## Abstract

**Background:**

For individuals who sustain a complete motor spinal cord injury (SCI) and rely on a wheelchair as their primary mode of locomotion, overground robotic exoskeletons represent a promising solution to stand and walk again. Although overground robotic exoskeletons have gained tremendous attention over the past decade and are now being transferred from laboratories to clinical settings, their effects remain unclear given the paucity of scientific evidence and the absence of large-scale clinical trials. This study aims to examine the feasibility of a locomotor training program with an overground robotic exoskeleton in terms of recruitment, attendance, and drop-out rates as well as walking performance, learnability, and safety.

**Methods:**

Individuals with a SCI were invited to participate in a 6 to 8-week locomotor training program with a robotic exoskeleton encompassing 18 sessions. Selected participants underwent a comprehensive screening process and completed two familiarization sessions with the robotic exoskeleton. The outcome measures were the rate of recruitment of potential participants, the rate of attendance at training sessions, the rate of drop-outs, the ability to walk with the exoskeleton, and its progression over the program as well as the adverse events.

**Results:**

Out of 49 individuals who expressed their interest in participating in the study, only 14 initiated the program (recruitment rate = 28.6%). Of these, 13 individuals completed the program (drop-out rate = 7.1%) and attended 17.6 ± 1.1 sessions (attendance rate = 97.9%). Their greatest standing time, walking time, and number of steps taken during a session were 64.5 ± 10.2 min, 47.2 ± 11.3 min, and 1843 ± 577 steps, respectively. During the training program, these last three parameters increased by 45.3%, 102.1%, and 248.7%, respectively. At the end of the program, when walking with the exoskeleton, most participants required one therapist (85.7%), needed stand-by or contact-guard assistance (57.1%), used forearm crutches (71.4%), and reached a walking speed of 0.25 ± 0.05 m/s. Five participants reported training-related pain or stiffness in the upper extremities during the program. One participant sustained bilateral calcaneal fractures and stopped the program.

**Conclusions:**

This study confirms that larger clinical trials investigating the effects of a locomotor training program with an overground robotic exoskeleton are feasible and relatively safe in individuals with complete motor SCI. Moreover, to optimize the recruitment rate and safety in future trials, this study now highlights the need of developing pre-training rehabilitation programs to increase passive lower extremity range of motion and standing tolerance. This study also calls for the development of clinical practice guidelines targeting fragility fracture risk assessment linked to the use of overground robotic exoskeletons.

## Background

There has been a growing interest for overground robotic exoskeletons over the past decade [1–8]. These overground robotic exoskeletons typically provide motorized assistance at the hips and knees via motors while the ankles and feet are generally assisted with dynamic ankle-foot orthoses. This assistance fully or partially generates and coordinates flexion and extension movements and moments at these joints to produce or assist with sit-stand transfers and overground walking. For individuals who are affected by sensorimotor impairments and rely on a wheelchair as their primary mode of locomotion, overground robotic exoskeletons figure among the most promising solutions to stand and walk although their effects and effectiveness remain unclear given the paucity of scientific evidence.

In this population, overground robotic exoskeletons can be used for standing and walking in the context of adapted physical activity programs offered during rehabilitation or in the community. In fact, based on the available evidence, adapted physical activity programs incorporating standing and walking with a robotic exoskeleton could potentially alleviate the development of musculoskeletal [9], cardiorespiratory [10–13], and endocrine-metabolic [9] secondary health conditions and complications. Hence, there is a need to develop and test such programs that integrate an overground walking component while also democratizing accessibility to the robotic exoskeleton, especially in publically-funded healthcare environments. However, before doing so and assessing such programs in terms of their efficacy and effectiveness, it is crucial to gain a better understanding of the factors that could potentially interfere in the process of participants’ recruitment and selection as well as in their attendance at the training sessions. To date, only few studies have reported their recruitment rate or identified the personal or environmental factors that interfered in the selection of potential participants, and even fewer have reported their attendance rate whenever a training program was offered [14–17]. Moreover, gaining a better understanding of the skill-acquisition process during overground walking with a robotic exoskeleton, and of a safe and well-tolerated progression is essential for planning future larger-scale interventional trials. To date, very few studies have precisely described the trajectory of change observed over the course of the training program, especially in regard to the number of therapist required and their level of physical assistance, the type of walking aid required during each training session, and the time needed to achieve autonomous control of the exoskeleton [18]. However, many studies have reported training-related measures (e.g., standing time, walking time, number of steps taken during a session) or performance-based measures (e.g., walking speed- or distance) that were typically measured only at the start, midterm, or end of the intervention [15]. Based on a recent systematic review incorporating 14 case-series or quasi-experimental studies using a robotic exoskeleton as an assistive device [2], mostly small heterogeneous group of individuals with complete and incomplete sensorimotor SCI (i.e., *N* ≤ 8 participants in 86% of the studies) who completed, using different models of overground robotic exoskeletons having various control modes (i.e., 5 different exoskeletons used with 4 different control modes), various training protocols encompassing a wide range of training sessions (range: 2 to over 100 training sessions) and frequencies (range: unspecified to 6 training sessions per week) offered at a single center have been investigated to date. Hence, stronger evidence continues to be needed to inform the development of future evidence-based adapted physical activity or neurorehabilitation training programs to be tested and compared in larger-scale interventional trials. Alongside, stronger evidence on the cardiorespiratory [11], musculoskeletal, balance, and cognitive requirements during overground walking with a robotic exoskeleton, for examples, is also needed to better understand the underlying mechanisms of intervention effects and to select the best comparators in these future trials.

The overall aim of the present study was to investigate the feasibility and safety of a new locomotor training program with a robotic exoskeleton offered to long-term manual wheelchair users with a spinal cord injury (SCI) living in the community. Specifically, the intent of the present study was to precisely determine the recruitment, attendance and drop-out rates, the learnability and detailed progression over the course of the training program (including the level of human assistance, the level of technical assistance, and the walking performance in terms of walking time, number of steps taken, and walking speed), and the safety. These attributes of the new locomotor training program could provide valuable information for the development of future larger-scale clinical trials investigating the effects or the efficiency of a locomotor training program with an overground robotic exoskeleton. These clinical trials are important to better understand if and how locomotor training programs with the robotic exoskeleton can alleviate secondary health conditions and complications, maximize functional abilities, or optimize psychological well-being, social participation, and life satisfaction among long-term manual wheelchair users living in the community.

## Methods

### Design

A single-group longitudinal prospective feasibility study.

### Participants

A sample of 14 adults with a motor complete SCI (ASIA Impairment Scale = A or B) who use a wheelchair as their primary mode of mobility were recruited (Table 1). To be included in the study, potential participants had to be at least 18 years of age, had been discharged from an intensive inpatient rehabilitation program for at least 18 months, resided in a community within a 75 km radius of the rehabilitation center, and communicated in either French or English. Potential participants who had previously underwent training with robotic exoskeleton for overground walking, with other nervous system damage aside from the SCI (e.g., multiple sclerosis), impaired skin integrity (e.g., pressure sores in areas in contact with the robotic exoskeleton), concomitant or secondary musculoskeletal impairments (e.g., lower extremity heterotopic ossification, rotator cuff tendinopathy), history of lower extremity fracture within the past year, unstable cardiovascular or autonomic system, cognitive or oral communication problems, or any other conditions that could restrict their ability to train walking ability or confound in other ways the results of this study were excluded. All potential participants were also screened by a research physiotherapist to rule out other potential standing- and walking-related exclusion criteria such as lower extremity passive range of motion limitations (hip flexion contracture ≥5°, knee flexion contracture ≥10°, and ankle dorsiflexion ≤ − 5° with knee extended), moderate-to-severe lower extremity spasticity (> 2 modified Ashworth score), inability to sit with hips and knees ≥90° flexion, and a standing tolerance test with full lower extremity weight-bearing of ≤30 min. Moreover, for participants to be fitted within the robotic exoskeleton, their height and pelvis width needed to range between 1.52–1.93 m and 30–46 cm, respectively, whereas the length of their thigh and lower leg segments also needed to range between 51 and 61.4 cm and 48–63.4 cm, respectively. Furthermore, a length discrepancy of no more than 1.3 and 1.9 cm was essential at the thigh and lower leg segments, respectively. Last, the participant’s body weight needed to be less than 100 kg. The main recruitment strategies implemented by the research team included contacting individuals with a complete motor SCI who previously participated in research projects and had agreed to be informed when a new study starts; posting flyers containing information about the study in key areas within the rehabilitation facility; advertising in the magazine of a non-profit organisation dedicated to the social and professional reintegration of individuals with spinal cord injury in the province of Quebec (i.e., www.moelleepiniere.com/en/our-publication/paraquad/); and educating physicians servicing individuals with SCI living in the community via the outpatient clinic at the rehabilitation facility about the study for them to refer potential participants to the research team. Alternative recruitment strategies included potential participants who directly contacted the research team to express interest in participating after having observed part of a training session during a visit to the rehabilitation facility; who saw a television show reporting on the research project (https://www.youtube.com/watch?v=1y1c6ynYySk); who discussed with a participant involved in or who had completed the study; or who had read comments about the study or seen videos of participants engaged in the study posted on social media (i.e., Facebook). The study was conducted at the Pathokinesiology Laboratory of the Centre for Interdisciplinary Research in Rehabilitation of Greater Montreal (CRIR) located at the CIUSSS du Centre-Sud-de-l’Île-de-Montréal–Site: Installation Institut de réadaptation Gingras-Lindsay-de-Montréal. All participants gave their written consent to participate after being informed of the study’s objectives and of the nature of their participation. The Research Ethics Committee of the Centre for Interdisciplinary Research in Rehabilitation of Greater Montreal approved the study (CRIR-1083-0515).

**Table 1:** Description of participants

Participant #	Sex	Age (years)	Height (m)	Weight (kg)	Body Mass Index (Weight/Height^2^)	Time since SCI/D (years)	Origin of SCI/D	ASIA-Motor Score (/100)	ASIA-Sensory Score (/224)	ASIA Impairment Scale (AIS)	ASIA Neurological level
1	F	26.7	1.61	61.4	23.7	2.2	Trauma	50	104	A	T6
2	M	28.4	1.78	73.9	23.3	5.1	Trauma	50	108	A	T6
3	M	63.1	1.85	96.0	28.0	8.3	Trauma	50	143	A	T10
4	M	32.2	1.92	91.2	24.7	8.0	Trauma	50	118	A	T6
5	M	42.9	1.8	66.6	20.6	14.4	Trauma	28	48	A	C6
6	M	51.5	1.67	61.9	22.2	31.4	Trauma	50	140	B	T6
7	M	43.8	1.8	107.2	33.1	3.4	Trauma	50	143	A	T10
8	M	35.3	1.87	67.9	19.4	8.6	Trauma	50	108	A	T6
9	M	38.1	1.6	64.3	25.1	6.9	Trauma	50	115	A	T9
10	M	27.2	1.7	56.2	19.4	4.2	Trauma	50	104	A	T4
11	F	31.1	1.6	63.7	24.9	1.0	Trauma	54	123	A	T8
12	F	39.4	1.68	75.5	26.8	4.7	Trauma	50	80	A	T3
13	F	51.9	1.62	58.5	22.3	5.2	Non-Trauma	50	96	A	T4
14	F	30.9	1.63	48.7	18.3	0.8	Trauma	50	106	A	T6
Mean		38.7	1.7	70.9	23.7	7.4		48.7	109.7		
Standard deviation		10.9	0.1	16.5	3.9	7.8		6.1	25.4		

*AIS* ASIA Impairment Scale, *ASIA* American Spinal Cord Injury Association, *A* No motor or sensory function is preserved below the neurological level, *B* Sensory function is preserved but no motor function below the neurological level, *C* Motor function is preserved below the neurological level, and more than half of the key muscles below the neurological level have a muscle grade < 3 out of 5 (manual muscle testing), *D* motor function is preserved below the neurological level, and at least half of the key muscles below the neurological level have a muscle grade of ≥3 out of 5, *E* motor and sensory function are normal

### Robotic exoskeleton

The wearable robotic exoskeleton EKSO™ (version 1.1) (Ekso Bionics, Richmond, CA, USA) is a ready-to-wear, battery-powered, motor driven, robotic pair of legs generating motion at the hip and knee joints in a properly sequenced manner. Each joint is independently controlled by different sensors linked to a small, portable, computerized control system attached to the flexible trunk module that also encompass the battery. Information gathered by over 35 different sensors (e.g., accelerometers, speed controllers, gyroscopes, pressure sensors) feed a decisional algorithm loop allowing manual wheelchair users with SCI to perform sit-stand transfers and walk. When walking with the robotic exoskeleton, each step is primarily commanded by combined forward and lateral bodyweight shifts toward the weight-bearing lower extremity before initiating the oscillation with the opposite lower extremity. The certified therapist can control numerous walking features (e.g., speed, step height, step length). The EKSO GT robotic exoskeleton weighs about 28 kg and can technically reach a maximal walking speed of 1.6 m/s. The EKSO GT robotic exoskeleton is approved by Health Canada for clinical use.

### Locomotor training program

Initially, participants attended two familiarization sessions over a one-week period that lasted about 45–60 min per session. During these familiarization sessions, participants were properly fitted with the EKSO GT robotic exoskeleton before performing balance, walking-related tasks (e.g., sit-stand transfers), and walking on short distances with visual and verbal feedbacks while the certified therapist actuated each step (i.e., *FirstStep* mode). As the participants’ level of proficiency increased during the familiarization sessions, they learned to safely ambulate with the exoskelon at a self-selected comfortable speed using their own walking aid (i.e., rolling walker or forearm crutches) while taking control of each step triggered via anterolateral body weight shifts (i.e., *ProStep* mode) under the direct supervision of a certified therapist. Following these two familiarisation sessions, participants began the six-week progressive locomotor training program administered by a certified therapist that encompassed a total of 18 training sessions (three sessions/week; 60 min/session). Depending on the level of each participant’s proficiency, on the participant’s tolerance, and on the activities planned for the session (e.g., instructions and basic training to initiate sit-stand transfers, walking and turning with forearm crutches), the workload was periodically adjusted using walking distance, duration, and speed parameter progressions [7]. After each session, all training parameters and other relevant information (e.g. total standing time, total walking time, and total number of steps) were recorded.

### Main outcome measures

The main outcome measures are the rate of recruitment of potential participants, the drop-out rate of participants enrolled into the study, the rate of attendance at training sessions, the progression in the ability to walk with the exoskeleton (i.e., standing time, walking time, number of steps taken per session, type of waking aid, number of therapists needed, level of assistance provided per session), the performance when walking with the exoskeleton at self-selected comfortable walking speed measured using the 10-m walking test (10MWT) [19] at the start (within the first 5 sessions) and end of the program, and adverse events (Table 2).

**Table 2:** Summary of the key outcome measures

Attendance		Learnability and Performance								Mobility Aid and Physical Assistance							Safety				
Subject ID	# of training sessions completed (/18)	Length of time spent standing upright (min/session)		Length of time spent walking (min/session)		Number of steps taken (steps/session)		Walking Speed (m/s)		First training session			Last training session			Use of controller (session achieved)	Shoulder pain or stiffness	Thumb Tendinitis (Adductor)	Knee instability	Pressure Drop (Request to sit)	Fracture (Ankle - Bilat)
										Level of assistance	Walking Aid	Number of PT needed	Level of assistance	Walking Aid	Number of PT needed						
		mean (SD)	[min;max]	mean (SD)	[min;max]	mean (SD)	[min;max]	Start	End												
1	18	52(12)	[19; 70]	30(9)	[8;45]	1240(492)	[297;2087]	0.16	0.29	3	F.C.	2	5	F.C.	1	15	☑	–	–	☑	–
2	18	49(6)	[41; 62]	31(9)	[13;49]	1169(412)	[420;2050]	0.19	0.30	3	F.C.	2	6	F.C.	1	15	–	–	–	☑	–
3	18	56(13)	[33;87]	30(7)	[19;41]	828(260)	[407;1262]	0.12	0.24	3	R.W.	2	4	F.C.	1	Not Achieved	–	–	–	–	–
4	17	54(11)	[29;75]	33(10)	[20;54]	1096(453)	[371;2148]	0.16	0.23	3	R.W.	2	5	F.C.	1	Not Achieved	–	–	–	–	–
5	14	37(11)	[18;51]	19(8)	[5;33]	588(293)	[114;1056]	–	0.20	2	R.W.	2	3	R.W.	2	Not Achieved	–	–	–	☑	–
6	18	53(9)	[29;63]	40(11)	[19;52]	1575(535)	[738;2272]	0.16	0.27	4	R.W.	1	6	F.C.	1	12	☑	–	–	–	–
7	18	56(9)	[34;71]	38(9)	[22;54]	1226(407)	[768;2101]	0.13	0.30	3	R.W.	2	4	R.W.	1	Not Achieved	☑	–	☑	–	–
8	1	51-	[51;51]	19-	[19;19]	455 -	[455;455]	–	–	3	R.W.	2	3	R.W.	2	Not Achieved	–	–	–	–	☑
9	18	45(14)	[18;60]	34(16)	[7;55]	1347(757)	[221;2397]	0.13	0.24	3	R.W.	1	6	F.C.	1	13	–	–	–	–	–
10	18	32(10)	[12;50]	21(9)	[6;40]	765(386)	[254;1555]	0.17	0.17	3	R.W.	2	4	R.W.	1	Not Achieved	–	–	–	☑	–
11	18	55(7)	[37;64]	41(11)	[19;53]	1504(491)	[601;2097]	0.16	0.29	3	F.C.	2	5	F.C.	1	12	–	–	–	–	–
12	18	57(6)	[38;65]	44(9)	[24;59]	1266(454)	[445;2093]	0.12	0.22	3	F.C.	2	5	F.C.	1	12	☑	☑	–	☑	–
13	18	43(13)	[17;62]	29(11)	[11;45]	1057(485)	[317;1702]	0.13	0.14	3	R.W.	2	4	F.C.	1	Not Achieved	–	–	–	☑	–
14	18	55(10)	[39;71]	42(12)	[22;58]	1711(614)	[567;2527]	0.15	0.28	3	R.W.	2	6	F.C.	1	8	–	–	–	–	–
Mean	17.6^a^	49.7		33.4^b^		1190^b^		0.15	0.25												
Standard deviation	1.1	12.7		12.5		561.4		0.02	0.05												

Level of Assistance:(1): Dependent: During dependent mobility, the participant is unable to help at all. The physical therapist - or another healthcare provider - will do all of the work

(2): Maximal Assist: The participant performs 25% or less of the work during mobility and the physical therapist provides the rest of the work

(3): Moderate Assist: The participant performs between 25% and 75% of the work necessary to move and the physical therapist provides the rest of the work

(4): Minimal Assist: The participant performs 75% of the work to move and the physical therapist provides the rest of the work

(5): Contact Guard Assist: The physical therapist needs to merely have one or two hands on the participant’s body, but provides no other assistance to perform the functional task. The contact is made to help steady the body or help with balance

(6): Stand-by Assist: The physical therapist does not touch the participant or provide any assistance, but he or she may need to be close by for safety in case the participant loses their balance or needs help to maintain safety during the task being performed

(7): Modified Independence: The participant can walk with the exoskeleton without any supervision, with the help of a walker or crutches

(8): Total independence: The participant can walk with the exoskeleton without supervision and the use of a walking aid

Walking Aid: *R.W.* Rigid walker and *F.C.* Forearm crutches

^a^Participant 8 was excluded

^b^Weighted average

### Statistical analysis

Descriptive statistics (i.e., mean, standard deviation) were calculated for all demographics and clinical characteristics as well as for all outcome measures. After a Shapiro-Wilk test confirmed the normality of the walking speed measures, the pre- and post-training walking speed measures were compared using a paired Student *t* test for repeated measures with the significance level set at *p* ≤ 0.05. These statistics were computed using SPSS statistic software version 17.0 (IBM Corporation, Armonk, New York).

## Results

### Recruitment

**Fig. 1:**
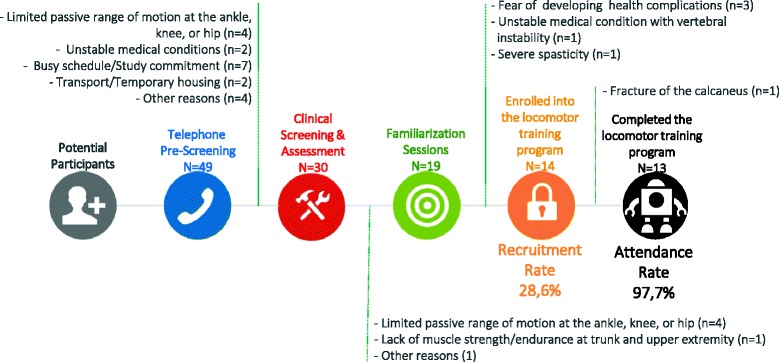
Summary of the key milestones of the project

A summary of the recruitment process, along with the number of participants who successfully completed each stage of this process and reasons for excluding potential participants, are illustrated in Fig. 1. A total of 49 individuals with a SCI contacted the research team by phone or via email to express their interest in participating in the research project during an overall 11-month enrollment period split into two phases: October to December 2015 (3 months) and May to December 2016 (8 months). In reality, the overall enrollment period per se was shorter (about 8 months) since the last participants needed to be recruited no later than November 1, 2015 and 2016, respectively. Upon completion of the pre-screening interview of potential participants over the phone (*N* = 49), 19 individuals with a SCI were excluded for different reasons based on the answers provided to specific screening questions during this initial step. Out of the 30 potential participants who underwent clinical pre-screening, 11 individuals with SCI were excluded for different reasons during this second step. Finally, out of the last 19 potential participants who underwent clinical screening and completed the familiarization sessions, five individuals with SCI refused to participate in the study during this last recruitment step. Hence, a total of 14 individuals with SCI were enrolled in the study (recruitment rate = 28.6%). The most common reasons for excluding potential participants were the presence of limited passive dorsiflexion range of motion at the ankle (*n* = 13/30; rate = 43.3%) as well as time, transportation, or accommodation constraints linked to the program requirements (*N* = 9/30; rate = 30%). The most common reason for refusing to participate was the fear of developing a complication as a result of ambulating with the robotic exoskeleton system after having tried the robotic exoskeleton (*n* = 3/5; rate = 60%).

### Attendance

Most participants (*N* = 11/14) completed all training sessions (attendance rate = 100%) whereas two participants were deprived of one (adjusted attendance rate = 94%) and four training sessions (adjusted attendance rate = 78%), respectively, since the program was temporarily suspended during the holiday season. Hence, the overall attendance rate was 97.9% (229 completed training sessions/234 planned training sessions). One participant was withdrawn from the study by the research team after one training session and was not accounted for in the attendance rate and learnability statistics (details provided in *Adverse Events* section). This was the only participant who dropped out of the study (*n* = 1/14; drop-out rate = 7.1%).

### Learnability and performance

**Fig. 2:**
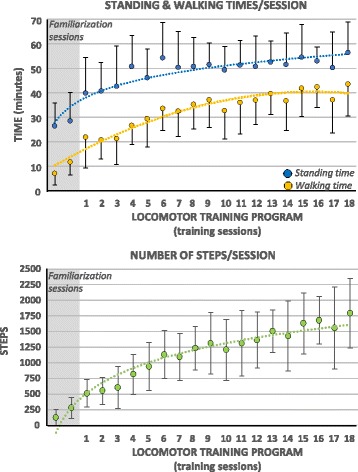
Group mean ± 1 SD of the standing time, walking time, and number of steps measured per session

**Fig. 3:**
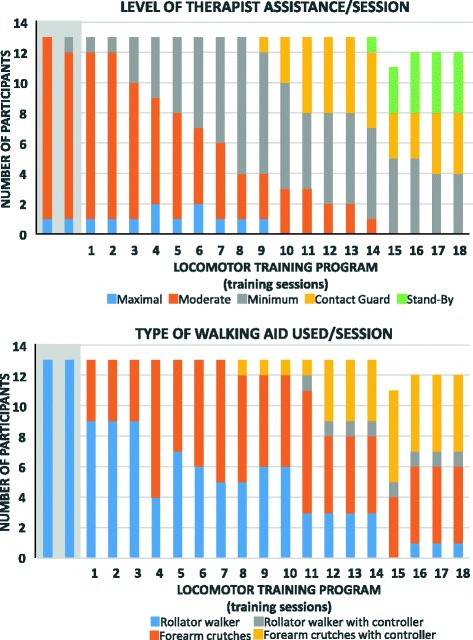
Description of the level of therapist assistance required and of the walking aid used during each session

A summary of the progression of the standing time, walking time, and number steps taken per session is illustrated in Fig. 2. On average, during the locomotor training program, the standing time, the walking time, and the number of steps taken per session were 49.7 ± 12.7 min, 33.4 ± 12.5 min, and 1190 ± 561 steps, respectively. Between the start (mean of sessions 1 and 2) and the end (mean of sessions 17 and 18) of the locomotor training program, the standing time, walking time, and number of steps taken per session progressed by 45.3%, 102.1%, and 248.7%. The majority of participants (*N* = 10/13) were already self-initiating their steps via lateral and anterior bodyweight shifts toward the weight bearing lower extremity (i.e., prostep mode) at the first session of the training program. Two additional participants reached this level at the second training session whereas another participant reached it at the 8th session of the training program. Most participants (*N* = 10/13) developed the ability to ambulate with Canadian crutches after 3.5 ± 3.3 training sessions whereas the other 3 participants continued to use a rollator walker throughout the training program (Fig. 3). Most participants (*N* = 11/13) needed moderate assistance provided by one physiotherapist at the start of the training sessions whereas the two participants needed minimal and maximal assistance, respectively (Fig. 3). Upon completion of the training sessions, one participant needed moderate, four participants needed minimal, and four needed contact guard assistance provided by one physiotherapist, whereas four participants needed a physiotherapist to stand-by while walking (Fig. 3). All participants needed moderate to maximal assistance provided by one physiotherapist for all sit-stand transitions throughout the training sessions. As for the walking speed, it increased significantly (*p* ≤ 0.0001; + 66.8%) between the start (mean ± 1 SD = 0.15 ± 0.02 m/s) and end (mean ± 1 SD = 0.25 ± 0.05 m/s) of the training program. These last results do not include the data of one participant with C6 tetraplegia as he only took a limited number of steps at a very slow pace and needed maximal assistance of the certified therapist during the first week (i.e., invalid result for the 10MWT).

### Adverse events

As mentioned previously, one participant was diagnosed with bilateral type I non-displaced fractures of the calcaneus after having completed the two familiarization sessions and the first training session. Uncertainties exist about the specific cause of the fractures. This participant was withdrawn from the study. Four participants reported exacerbation of pre-existing shoulder pain, stiffness, or discomfort whereas one participant developed soreness at the thumb over the course of the locomotor training program. This finding was unexpected as no previous study reported on upper extremity pain, stiffness, or discomfort. One participant failed to report a previously complete anterior cruciate ligament tear to the research team and developed severe knee hyperextension at the 10th training session that was solved by blocking knee extension to − 3° thereafter. Six participants experienced orthostatic hypotension with systolic blood pressure drops of ≥ 20 mmHg during a training session. No participant developed any soft tissue or skin problem nor fell during the locomotor training program. For the three certified trainers involved in the intervention, no adverse effect was documented. Last, a battery failure and a hip joint bearing assembly failure were directly linked to the robotic exoskeleton itself over the course of the locomotor training program.

## Discussion

### Recruitment

The recruitment rate reached in this preliminary study (28.6%) was acceptable although it remains relatively low considering the number of potential participants (*N* = 49) who initially expressed their interest in participating into the proposed study and completed the different steps of the recruitment process. Moreover, considering that the study was conducted in a rehabilitation center hosting an ultra-specialized SCI rehabilitation program servicing the western part of the Province of Quebec, the fact that numerous strategies were implemented to overcome potential barriers (e.g., a dedicated research professional in charge of the recruitment, multiple recruitment strategies implemented, telephone pre-screening interview to minimize the number of visits, free parking, free training sessions), and that all participants were allocated to the locomotor training program with the robotic exoskeleton, a higher recruitment rate was anticipated (i.e., ≥50%). Nonetheless, this recruitment rate is 1.7 times greater than the one reported in other feasibility studies investigating locomotor training programs with a robotic exoskeleton in individuals with complete or incomplete SCI in England (17%) [15] and Germany (12%) [18]. Only one recent multi-center study investigating a single training session with a self-stabilizing robotic exoskeleton in individuals with SCI has reached a recruitment rate near 50% (i.e., 20 participants recruited among 46 screened for eligibility) [17]. Nonetheless, the recruitment rate of the present study compares relatively well to the rates reached in other feasibility studies investigating various task-specific gait-training programs offered to relatively homogeneous samples of individuals with neurological impairments (e.g., stroke = 6.7% [20], Parkinson disease = 11% [21]). In the present study, the most important reason (16 out of 35 potential participants = 46%) for not qualifying for the training program was due to musculoskeletal impairments with the leading cause being a reduced passive range of motion at the ankle, knee, or hip. For the same reason, other preliminary studies have excluded up to 77.8% of potential participants (7 out of 9 potential participants excluded) [16]. The second most important reason was linked to time constraints (7 out of 35 potential participants = 20%). Contrary to other preliminary studies [16, 22], transportation did not emerge as a major barrier to the recruitment process nor the dropout rate in the present study since only two potential participants based their decision on this criterion (2 out of 35 potential participants = 5.7%). Taking these reasons together, developing a home-based pre-training program with indirect supervision of a therapist that would target gains in passive range of motion at the lower extremity and progressive standing time prior to initiating the locomotor training program may be warranted.

Different strategies may need consideration to optimize recruitment rate and facilitate attendance in future clinical trials [e.g., offering training sessions during the evening and weekend; offering training sessions away from the main rehabilitation center affiliated with the project (e.g., other rehabilitation centres, community-based physical activity centers, living labs in shopping malls); adjusting the training schedule to best match participants’ availability with a minimum of two training sessions per week; proposing temporary housing alternatives for potential participants living further away who demonstrate an interest in participating]. Last, it is important to highlight that the recruitment rate may have been lower if potential participants had had a chance to be allocated to an alternative experimental group undergoing a different training program or a control group with no training program.

### Attendance

The attendance rate reached in the present preliminary study (97.9%) was excellent. The high attendance with respect to the scheduled training sessions confirms the commitment of the participants who engaged into the locomotor training program. This is further supported by the fact that only one participant dropped out of the program following an adverse event (i.e. calcaneus fracture). Hence, a completion rate of 92.9% (*n* = 13 participants/14 participants) was reached and is greater than the 50% documented in another feasibility study proposing a comparable program [15]. The importance of the familiarization sessions needs to be highlighted since 5 out of the 19 participants (26.3%) decided not to engage into the locomotor training program at that time. Although not formally documented, these familiarization sessions allowed the research team, to some extent, to further screen potential participants who were hesitant to engage into the locomotor training program and potential participants to take an informed decision about their commitment based on a lived experience. Additionally, it supports the relevance of adopting a flexible approach when scheduling the training sessions to accommodate all stakeholders, especially the participants. Since the training sessions involved no or very limited socialization with individuals with similar sensorimotor impairments and functional disabilities (i.e., individualized approach), aside from the interaction with one or two therapists, the commitment of participants to complete the training session and, to some extent, their acceptance of the new technology, especially with regard to its perceived usefulness and ease-of-use, is also established.

### Learnability and performance

Individuals with a complete motor SCI demonstrated a capability to quickly learn to ambulate overground with a robotic exoskeleton. Overall, the standing and walking time (including the number of steps/session) progressed at a faster rate during the first half than during the second half of the locomotor training program. Overall, the participants stood and walked at least 30 and 20 min, respectively, at the first training session which is recommended in clinical practice to anticipate beneficial effects among long-term manual wheelchair users with a spinal cord injury [23, 24]. The level of therapist assistance also rapidly decreased over the course of the locomotor training program with most participants requiring no more than minimal assistance after the 8th and 9th training sessions (halfway into the program) and only contact guard or stand-by assistance by the end of the program when walking. Additionally, most participants walked with forearm crutches, with or without the use of the self-controller that allows the user to drive few basic functions of the robotic exoskeleton (e.g., initiation of the first step, continuous walking in ‘*prostep’* mode, and stops), by the end of the program. Overall, the learnability trajectory, illustrated for the first time in the present study using measures systematically collected at each training session, compares to some extent with the ones reported using only pre- and post-intervention measures in previous study using a similar or different robotic exoskeletons [3, 16, 18, 22].

The learning process also may have been facilitated by optional distinct auditory feedbacks automatically generated when the participant respectively reached the lateral and forward body weight shift targets required prior to initiating steps, especially early on during the learning stage [25]. Moreover, although not formally assessed in the present study, some participants periodically filmed their performance, especially at the beginning of the study, to facilitate their learning and complement the therapist’s subjective feedback (i.e., visual feedback-induced performance improvement) [26]. Hence, in addition to the adjustability of some exoskeleton parameters (e.g., reducing body shift amplitudes to initiate steps, reducing step height, increasing step length), numerous clinical strategies (e.g., reducing level of human assistance, changing walking aid) are also possible to adjust the level of challenges during overground walking with the robotic exoskeleton as the participant’s level of proficiency improves. Maintaining a level of challenge during learning is also known to positively impact a participant’s level of motivation and attendance, both of which are crucial in the context of any clinical trial in which participants are assigned to receive an intervention [27].

As for the performance, the walking speed was found to increase significantly between the start and end of the training program. In fact, the mean walking speed reached in the present study (i.e., 0.25 ± 0.05 m/s) is similar to the weighted mean gait speed of 0.25 ± 0.14 m/s reported in a recent meta-analysis investigating a heterogeneous group of individuals with a complete SCI who completed, with different models of overground robotic exoskeletons, various training protocols encompassing a wide range of training sessions [2]. Nonetheless, reaching faster walking speed after 18 sessions may still be possible with additional training since able-bodied adults, who have completed basic training with the robotic exoskeleton, reach on average a self-selected comfortable walking speed of 0.38 ± 0.09 m/s when they were asked to avoid all voluntary muscular contraction of their lower extremities (i.e., passive walking) [28].

### Safety

Among all participants, one serious adverse event occurred during the study. One participant sustained bilateral type I non-displaced fracture of the calcaneus after completing the two familiarization and the first training sessions. Although uncertainties exist about the specific cause of the fractures, both the fragility fracture risk of the calcaneus and the elevated vertical ground reaction force, known to reach about 36% ± 15% of the bodyweight at heel strike when walking with an overground robotic exoskeleton [29], are potential explanatory factors. This participant was withdrawn from the study and referred to the medical team until the fractures were healed. These fractures occurred even though the screening process was thoroughly completed by an experienced research physiotherapist and the minimal standing time tolerance (i.e., ≥30 min), recommended by the manufacturer of the exoskeleton, was verified. Unfortunately, another preliminary study also reported a comparable fracture of the talus during a locomotor training program with another overground robotic exoskeleton [15] whereas a review recently suggested an overall incidence rate of bone fracture of 3.4% [3]. The fact that some studies have predominantly included individuals with recent SCI (≤ 1 year), a time period during which bone mineral density declines at the L/Es and distal vertebrae (i.e., infralesional osteoporosis) may not have yet stabilized at levels significantly below those of age and gender-matched able-bodied individuals [30], may explain why this risk may have been underestimated [16]. Further investigation will be needed to explore all potential causes to implement additional screening elements for severe osteoporosis into the process (e.g., fracture risk stratification algorithms for adults with SCI [31], threshold for bone mineral density or architecture at the ankle and foot) and to develop solutions addressing the complex challenges linked to physical activities performed in standing position in individuals with SCI in the future. Other minor adverse events, predominantly linked to exacerbation of pre-existing (*N* = 4) or the development of new (*N* = 1) musculoskeletal-related non-debilitating pain at the upper extremity, were also documented (*n* = 5/14; 35.7%) over the course of the locomotor training program. Yet, all these participants opted to continue the training sessions while exploring personalized solutions to alleviate or even eliminate pain over the course of the program (e.g., increased number and duration of rest periods during sessions; cushioning at the handle of the walking aid; recommendation of stretching exercises post-training; use of nonsteroidal anti-inflammatory drugs) with the certified trainer(s). Unexpectedly, no exoskeleton-related skin or soft tissue issue was observed in the present study although it affected up to 50% of participants in previous studies and typically leads to interruptions of the intervention or withdrawal of participants from the studies [15]. All the above-identified risks remain impossible to eliminate, warrant thoughtful consideration, and should be carefully explained within the informed consent form along with the strategies implemented to minimize or overcome them. Finally, two events linked to the robotic exoskeleton itself (device malfunction) occurred over the course of the locomotor training program. The first event was a battery failure that required its replacement while the second event was a mechanical problem with a hip joint bearing assembly malfunction due to a damaged bolt holding the proximal and distal joint components together. In both cases, the problems were solved within a 48-h period with the prompt assistance of the company’s customer service department and had minimal impact on the conduct of the study.

### Limits of the study

Limitations in the present study were the small sample size of relatively homogeneous participants recruited at a single site as well as the absence of a control group. Uncertainties about the best research design and outcome measures to adopt in future clinical trials continue. Because the study only included long-term manual wheelchair users with a chronic SCI living in the community, the generalizability of the results beyond this reference population, such as in ambulatory individuals with an incomplete SCI, requires caution. Prudence is also suggested when inferring about participants’ acceptance and satisfaction, particularly when addressing attendance and learnability, as this dimension was not reported. Hence, the findings of the present study should be considered preliminary, but it is anticipated that they will stimulate interest in conducting future larger-scale level I or II clinical trials investigating the efficacy or effectiveness of locomotor training programs with an overground robotic exoskeleton in long-term manual wheelchair users.

## Conclusion

This study reinforces what other pilot studies have shown and confirms that a locomotor training program with an overground robotic exoskeleton under the direct supervision of a certified therapist is feasible and relatively safe in long-term manual wheelchair users with complete motor SCI. This finding is expected to stimulate interest in conducting future Level I and II large clinical trials investigating, for example, the physical and psychological health effects or the cost-effectiveness of a locomotor training program with an overground robotic exoskeleton in this population. While doing so, strategies may need to be implemented to overcome potential challenges related to recruitment rate and minor safety issues. In fact, this study now confirms the relevance of developing pre-training rehabilitation programs to optimize passive lower extremity range of motion and standing tolerance to optimize the recruitment rate and safety, respectively. This study also calls for the development of clinical practice guidelines targeting fragility fracture risk assessment linked to the use of overground robotic exoskeletons.

## Abbreviations

ASIA: American Spinal Injuries Association
SCI: Spinal Cord Injury
